# Reprogramming Metabolism of Macrophages as a Target for Kidney Dysfunction Treatment in Autoimmune Diseases

**DOI:** 10.3390/ijms23148024

**Published:** 2022-07-21

**Authors:** Feng Tian, Hui Chen, Jianmin Zhang, Wei He

**Affiliations:** 1 Department of Immunology, CAMS Key Laboratory T Cell and Cancer Immunotherapy, Institute of Basic Medical Sciences, Chinese Academy of Medical Sciences and School of Basic Medicine, Peking Union Medical College, State Key Laboratory of Medical Molecular Biology, Beijing 100005, China; 1721020392@stu.cpu.edu.cn (F.T.); chenhui_1980@126.com (H.C.); 2 Haihe Laboratory of Cell Ecosystem, Tianjin 100730, China

**Keywords:** macrophage, autoimmune disease, chronic kidney disease, metabolism

## Abstract

Chronic kidney disease (CKD), as one of the main complications of many autoimmune diseases, is difficult to cure, which places a huge burden on patients’ health and the economy and poses a great threat to human health. At present, the mainstream view is that autoimmune diseases are a series of diseases and complications caused by immune cell dysfunction leading to the attack of an organism’s tissues by its immune cells. The kidney is the organ most seriously affected by autoimmune diseases as it has a very close relationship with immune cells. With the development of an in-depth understanding of cell metabolism in recent years, an increasing number of scientists have discovered the metabolic changes in immune cells in the process of disease development, and we have a clearer understanding of the characteristics of the metabolic changes in immune cells. This suggests that the regulation of immune cell metabolism provides a new direction for the treatment and prevention of kidney damage caused by autoimmune diseases. Macrophages are important immune cells and are a double-edged sword in the repair process of kidney injury. Although they can repair damaged kidney tissue, over-repair will also lead to the loss of renal structural reconstruction function. In this review, from the perspective of metabolism, the metabolic characteristics of macrophages in the process of renal injury induced by autoimmune diseases are described, and the metabolites that can regulate the function of macrophages are summarized. We believe that treating macrophage metabolism as a target can provide new ideas for the treatment of the renal injury caused by autoimmune diseases.

## 1. Introduction

Chronic kidney disease (CKD) is a progressive organic disease characterized by persistent alterations in kidney structure, function, or both with a severe impact on the health of the individual [1,2]. In the past decades, the number of patients with chronic kidney disease has significantly increased at a fast rate around the world. According to the 2019 Global Burden of Disease study, kidney dysfunction was ranked 18th in the list of the global number of deaths and percentage of disability-adjusted life-years (DALYs) in 2010 but rose quickly to 9th in 2019 [3,4,5]. Taking a panoramic view of all risk factors, not only diabetes and hypertension but also autoimmune diseases, especially lupus erythematosus, are the main causes of kidney failure. Approximately 50% of systemic lupus erythematosus (SLE) patients suffer from kidney disease [6,7]. Monitoring and protection of renal function have always been the focus of the treatment of autoimmune diseases. Under the continuous impact of various injury factors, the ultimate progression of CKD is renal fibrosis, scar tissue formation, and renal failure, and most CKD patients progress to end-stage renal disease (ESRD). Essentially, the different kidney disease stages are accompanied by the inflammatory infiltration of different compositions [8,9,10].

Macrophages have long been known as all-rounders in kidney injury repair and inflammation and are associated with the progression of kidney fibrosis [11,12]. Macrophages are the general term for a class of complex cells derived from an important part of the mononuclear phagocytic system, and they are characterized by their heterogeneity and plasticity [13,14]. Initially, researchers identified a group of cells with powerful phagocytosis and clearance, which were named macrophages based on the Greek “big eater” [15,16]. Subsequent research revealed that the versatility of macrophages is critically dependent on the local environment, and plays an important role in kidney damage and repair; they mainly remove pathogens and repair damaged tissue under certain conditions, further promoting tissue damage [17,18,19,20]. There are two main sources of renal macrophages, kidney-resident macrophages and monocyte-derived macrophages. Lineage tracing studies proposed that kidney-resident macrophages predominately originate from the yolk sac erythro-myeloid progenitors (EMP) and hematopoietic stem cells (HSC), and monocyte-derived macrophages infiltrate the kidney under inflammatory conditions. Macrophages from different sources together with other immune cells maintain the immune microenvironment of the kidney [18,21]. Some of the latest views proposed in this area have enunciated the role of macrophage plasticity and multifunction during the progression of kidney inflammation and renal fibrosis [13,22,23]. However, the role and functional changes of macrophages in the progression of the renal burden caused by autoimmune disease still need to be elucidated. In this review, we discuss the varied roles of macrophages in renal pathology caused by autoimmune disease and consider a therapeutic strategy to target macrophage-dependent kidney damage.

## 2. The Key Point of Macrophage Multifunction: Heterogeneity and Plasticity

**Figure 1:**
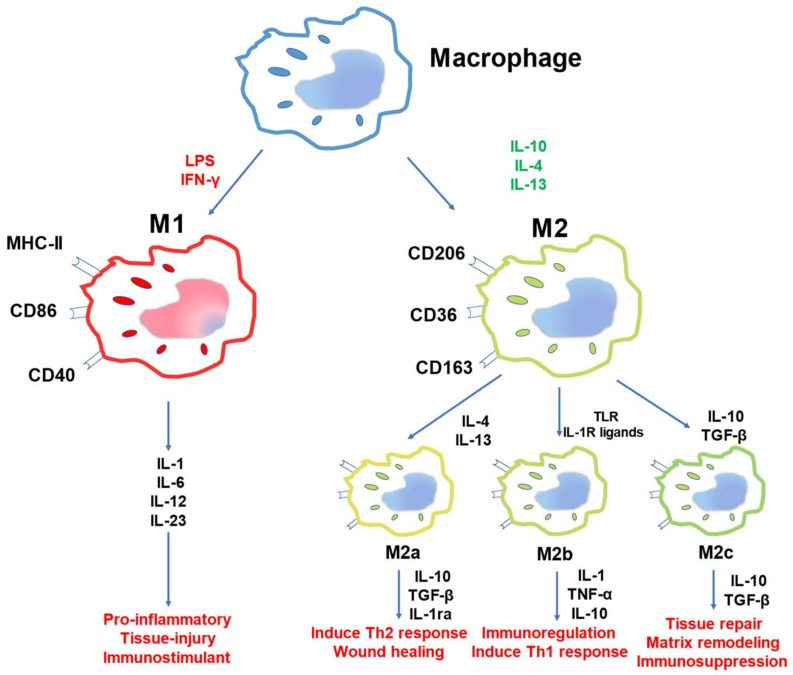
The polarization of macrophages. Inflammatory signals, such as IFN-γ and LPS, can polarize macrophages to the M1 phenotype, which contributes to proinflammation, tissue injury, and immunostimulation. M2 phenotype subsets are polarized by a variety of signals. Currently, M2 macrophages are divided into three categories, M2a, M2b, and M2c, which have immunoregulatory and tissue remodeling functions.

Macrophages consist of various tissues of our body, some of which are long-lived, tissue-resident macrophages that settle in tissues and organs, including in the kidneys [24,25,26,27]. Under healthy conditions, macrophages can be phenotypically polarized by surrounding microenvironment stimuli and signals to mount specific functional programs, even more so under the background of continuous tissue damage when the normal microenvironment is disrupted by many factors such as pathogens, cellular damage, immune responses, hypoxia, and tissue repair processes [28,29,30]. Macrophages can be phenotypically polarized to mount specific functional programs by surrounding microenvironment stimuli, such as pathogens, cellular damage, immune responses, hypoxia, and tissue repair processes [31,32,33]. Typically, two common phenotypes are used to represent two extreme states of macrophages, named M1 and M2 (Figure 1).

M1, the proinflammatory phenotype, plays an important role in the TH1-like immune response accompanied by the production of large amounts of proinflammatory cytokines. M1 macrophages, also known as classically-activated macrophages, activated by lipopolysaccharide (LPS), IL-1α, interferon γ (IFN-γ), and tumor necrosis factor (TNF). Thus, the active M1 cells produce an abundant number of proinflammatory factors, such as IL-1, IL-6, IL-12, and IL-23, and many chemokines such as CXCL1, CXCL3, CXCL5, CXCL8, CXCL9, CXCL10, CXCL11, and CXCL13, which induce early phases of healing and have high antigen presentation capacity [34,35,36]. A new point of view is demonstrated based on traditional activation methods: macrophages can be activated and polarized to the M1 phenotype through the miR-19b-3p/SOCS1 axis, which is initiated by the secretion of miR-19b-3p by tubular epithelial cells (TECs). Tubulointerstitial inflammation is derived from the initial injury of tubular epithelial cells (TECs), which is a common characteristic of CKD [37].

M2 macrophages, alternatively-activated macrophages, are polarized by the colony-stimulating factor (M-CSF), IL-4, IL-10, and IL-13, and are strong inhibitors of kidney damage through the suppression of inflammation and promotion of tissue repair. M2 macrophages are much more complex than M1 macrophages [38]. Thus far, M2 can be classified into three subpopulations, M2a–M2c, according to the phenotype and function [39,40].

M2a macrophages are induced by IL-4 and IL-13. They can secrete IL-10, CCL13, CCL17, and CCL22 and contribute to anti-inflammation through a TH2-like immune response and promote wound healing and induce tissue fibrosis [41,42,43]. M2b macrophages are stimulated by immune complexes and Toll-like receptors (TLR) and/or IL-1R ligands, participate in immunoregulation, and contribute to TH2-like activation. Recently, research showed that M2b macrophages could suppress the proliferation and migration of cardiac fibroblasts (CFs) by inhibiting the mitogen-activated protein kinase (MAPK) signaling pathway in cardiac fibroblast activation [44,45,46]. IL-10, transforming growth factor-β (TGFβ), and glucocorticoids can induce the differentiation of M2c macrophages, which play important roles in immunosuppression, matrix remodeling, and tissue repair [47,48,49].

Macrophages can also be regulated directly by external damaging factors. Calcium oxalate (CaOx) is the main component in kidney calculi, causing tubular damage and inflammatory responses. Aryl hydrocarbon receptor (AhR) is a ligand-activated transcription factor involved in the immune and inflammatory response, which is widely expressed in multiple cell types and is involved in the regulation of fundamental cellular processes [50,51]. Accumulating evidence demonstrates the AhR is the keeper of the physical and immunological barriers present at the mucosal surface’s sites [50]. However, the overexpression of AhR on Th17 cells indicates that it may have the opposite function in autoimmunity, which increases the risk of environmental factors on the susceptibility to infection and the development of autoimmune diseases. Zhang et al., detected methylation levels of AhR-related genes in 122 RA patients and 123 healthy controls by using the Illumina HiSeq platform [52]. The results show that compared with the control group, the *AHR* methylation level in RA patients was significantly higher, while the *AHRR* methylation level was abnormally reduced, which means the dysfunction of the AhR pathway’s regulation.The dynamic phenotypic balance of M1/M2 can be destroyed by the AhR-miR-142a-IRF1/HIF-1α pathway. Yang et al., demonstrated that the polarization of M1 macrophages reduced the deposition of crystals, and the polarization of M2 macrophages promoted the elimination of crystals through phagocytosis when CaOx was accumulated [53]. However, as mentioned above, the phenotype of macrophages is dynamic, which makes it difficult and complex to distinguish between these diverse functional populations. It is worth noting that polarization only represents the two extremes, and the reality is much more complicated.

### 2.1. Macrophage Plasticity

To perform different functions, macrophages respond to different signals they receive, including but not limited to microbial invasion, tissue damage, and adaptive immune responses.

The M1-to-M2 phenotypic switch may occur with the whole progression of the disease, which means that macrophages might undergo a further phenotypic change in the microenvironment [54]. Some evidence has identified this change, such as macrophage populations being polarized into the M1 phenotype in the early stage of a unilateral ureteral occlusion (UUO) model (by day 3); in that model the expression levels of granulocyte-macrophage colony-stimulating factor (GM-CSF), macrophage colony-stimulating factor (M-CSF), and GM-CSF were increased. This increased GM-CSF notably promotes the recruitment and proliferation of mononuclear phagocytes. By day 5, inflammatory M1 switched to a wound-healing M2 phenotype in the presence of M-CSF and prevented the further recruitment of monocytes/proliferation of resident macrophages [55]. M1 to M2 phenotypic shift is also promoted by GM-CSF expression and promotes the resolution of inflammation. In addition to the consistent expression of GM-CSF, the responsiveness of mononuclear phagocytes to M-CSF is reduced, thus preventing further recruitment of monocytes [56].

### 2.2. Macrophage Diversity

The complex and diverse cell sources also contribute significantly to the functional diversity of macrophages. For a long time, adult tissue-resident macrophages were thought to be continuously renewed by monocytes derived from bone marrow progenitor cells. However, there is evidence indicating that some tissue-resident macrophages arise from embryonic precursors before birth. These macrophages can self-renew and differentiate into M2 macrophages locally, contributing to anti-inflammation and tissue repair [57]. The complexity of cell-type origin and composition poses enormous challenges to the therapeutic options targeting macrophages. There is an urgent need to expand our understanding of the relationship between macrophage types and their matching functions [58]. The application of Single-Cell ProtEomics by Mass Spectrometry (SCoPE-MS) provides an efficient tool for the comprehensive cell decomposition of tissues. Some SCoPE-MS data showed proteins changed gradually across a continuous spectrum between M1 and M2-polarized primary human macrophages, and then various phenotypes of macrophages were classified by enriching different representative proteins [59]. Technical exploration allows researchers to characterize macrophage heterogeneity that emerges during cell differentiation and provides additional options for exploring regulatory interactions.

As we explore this unknown world, each additional dimension will allow us to understand more information and will make the description of things more in-depth and precise. Likewise, the advent of single-cell omics has led us to understand that the dots on a two-dimensional line (bulk sequencing) are not the same, i.e., heterogeneity between cells, giving us greater insight into the identity and state of cells within the tissue. We can better classify and analyze cells through two-dimensional means. However, our tissues or organs are three-dimensional, and single-cell omics lack spatial information, especially when various microenvironments play a decisive role in the fate and function of cells. The information brought to us by single-cell omics is limited or even inaccurate. Spatial omics technology has improved in response to proper time and conditions. Spatial mapping of all cells in healthy/obese mouse/human livers by integrating the single-cell cellular indexing of transcriptomes and epitomes by sequencing (CITE-Seq), single-nucleus sequencing, spatial transcriptomic, and spatial proteomic techniques revealed that the hepatic macrophage microenvironment is conserved and distinctive in different species. CITE-Seq makes up for the spatial information lost by single-cell sequencing, providing new information, and allowing us to understand the function and fate of every single cell in the tissue from a three-dimensional perspective [59]. Through the application of this technology, the spatial maps of mouse and human livers were displayed, the characteristics and microenvironment with the inclusion of Kupffer cells in the liver were described in detail, the conservation of and differences in macrophages in different species were revealed, and the discovery of a conserved cellular pathway associated with cell development and maintenance was discovered. Some subsets may be missed by using traditional flow cytometry to detect cell surface markers. A specific solution is provided by fusional modern technologies, such as integrating bulk tissue transcriptomics, single-cell RNA sequencing, and paired blood exchange to resolve myeloid cell heterogeneity in the UUO model, creating a single-cell atlas. By this hybrid method, the author identified five different macrophage clusters and matched their role in the development of kidney disease [60].

A detailed taxonomy and understanding of a macrophage’s plasticity can enhance our awareness of the role of macrophages in kidney injury and repair.

## 3. Metabolic Signature of Macrophage Responses

As the material basis and energy source, metabolism plays an irreplaceable role in the cell lifecycle. Macrophage polarization accompanies metabolic reprogramming and has emerged as a critical factor regulating various cell immune responses [61]. Macrophages with different phenotypes have different metabolic characteristics, which are illustrated herein in two significant cases. Glucose is the preferred energy source of M1 macrophages. However, alternatively-activated M2 macrophages prefer to use fatty acids as fuel for cellular behavior and activity [62]. The metabolic characteristics of M1 macrophages can be reasonably explained. More specifically, in the early stage of infection, the affected tissue microenvironment changes under the influence of factors, such as injury and infection. At this time, as part of the innate immune system, macrophages actively respond to this change by performing phagocytosis and initiating signaling; these processes require a large energy supply, and glucose metabolism is the most direct and efficient source in this process. However, the increased glucose metabolism certainly results in a relatively insufficient supply of oxygen. Therefore, positive regulation is generated to promote glucose transport and tolerance in a hypoxic environment by activating factors, such as hypoxia-inducible factor 1α (HIF1α), so that cells can better play a proinflammatory role and activate other immune cells to allow the body to fight infection as soon as possible [63,64]. Indeed, enhanced metabolism also provides the material basis for cellular inflammatory responses, which are important not only for anabolic or energy purposes but also for maintaining inflammatory responses [65,66].

**Figure 2:**
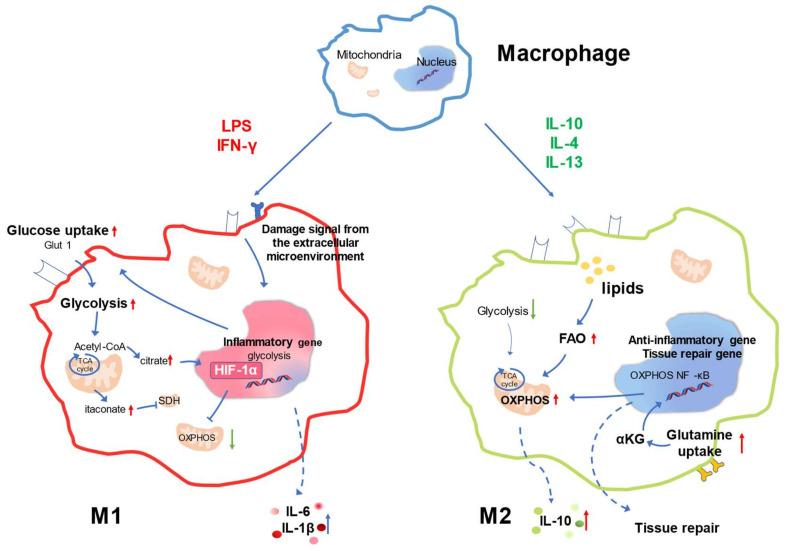
Metabolic reprogramming in macrophage polarization to proinflammatory or alternatively-activated macrophages. Proinflammatory polarization is accompanied by enhanced glycolytic metabolism (**left**), and OXPHOS, FAO, and glutamine metabolism are associated with alternatively-activated macrophages (**right**).

Different from M1, at the other extreme, alternatively-activated M2 has different characteristics. In M2-like macrophages, the TCA cycle is relatively intact, and many endogenous metabolites, such as citric acid and itaconic acid, play critical regulatory roles in M2 macrophages. In addition, the immune response of M2 macrophages is also regulated by amino acids and lipids and their metabolites (Figure 2) [13,66,67,68].

## 4. Changes in Macrophage Metabolic Pathways in the Injured Kidney of Autoimmune Disease

The specific metabolic characteristics of M1 and M2 are described in detail in some review articles, and will not be repeated in this article. The following will additionally describe the metabolic characteristics of macrophages in autoimmune diseases, supplement some of the latest findings, and summarize the implications for treating autoimmune diseases [13,69,70,71,72,73].

### 4.1. Regulation of Glucose Metabolism

Glucose is the main source of cell energy and is closely related to cell function and state. There is no doubt that the polarization of macrophages is often the result of changes in glucose metabolism. A variety of factors affecting macrophage polarization have gradually been discovered in recent years. Toll-like receptors (TLRs) are a class of important protein molecules involved in nonspecific immunity (innate immunity), which recognize molecules with conserved structures derived from microorganisms and activate immune responses [74]. TLR4 mainly recognizes Gram-negative bacterial lipopolysaccharide (LPS) and heat-shock proteins (HSP) released by necrotic cells of the host. Recent research has highlighted that the metabolism of macrophages is remodeled when the TLR of macrophages receives the message from LPS [75]. Macrophages rapidly increase glucose uptake and promote tricarboxylic acid (TCA) cycle volume in response to inflammation. Moreover, Lauterbach et al., found that TLR signaling enhances histone acetylation by directly increasing glucose production to acetyl-CoA through the adaptor proteins MyD88 and TRIF, ultimately enhancing the expression of a specific gene associated with proinflammatory responses. Based on the interrelation between glucose metabolism and histone acetylation, they further suggested that the IL12-acetylation axis could be a critical player in the progression of diabetes, and type 1 diabetes is considered an autoimmune disease [76].

### 4.2. Regulation of Glutamine Metabolism

Glutamine also plays a crucial role in immune regulation and is involved in cell proliferation and functional maintenance. Modulating glutamine metabolism could be an attractive strategy for harnessing macrophage-mediated immune responses. α-ketoglutarate (αKG) is produced by glutaminyls and promotes M2 activation through Jmjd3-dependent metabolic and epigenetic reprogramming. Simultaneously, suppressing M1 macrophages’ proinflammatory responses accompanies the accumulation of αKG. In addition, M1 activation can be restricted by αKG through the NF-κB pathway via PHD-mediated posttranslational modification of IKKβ. Different metabolic pathways provide different types of material and energy for macrophages with different functions. All these findings support the intervention of key nodes in these pathways and may provide important information for the development of combination therapies [77].

### 4.3. Regulation of Lipid Metabolism

As a part of metabolism, the LPS stimulation signal also changes the lipid metabolism of macrophages and then makes macrophages adapt to new functions and induces the production of inflammatory mediators [78]. Endogenous oxidized lipids simultaneously induce OXPHOS and aerobic glycolysis when macrophages revive the stimulated LPS. At the same time, endogenous oxidized lipids promote the hyperproduction of IL-1β in M1 through 1-palmitoyl-2-arachidonyl-sn-glycero-3-phosphorylcholine (PAPC) (also known as oxPAPC), which functions together with PAMPs to elicit immune responses [79]. Most recently, researchers found epigenetic changes upon prolonged exposure to oxidized low-density lipoprotein (oxLDL), which then drives the formation of proinflammatory macrophages [80]. Interestingly, polyunsaturated fatty acids (PUFAs), as one of the fatty acids, have been shown to play essential roles in inflammation regulation. In general, unsaturated fatty acids have a more active molecular structure than saturated fatty acids. As a part of PUFA, docosahexaenoic acid (DHA) has shown anti-inflammatory effects on chronic inflammatory disease [81]. DHA acts directly on monocytes to promote autophagosome formation, facilitating the polarization of U937 cells, which are the human monocytic cell lines polarization into M2-like macrophages via an autophagic mechanism through the p38 MAPK signaling pathway. Lipids are not only responsible for the energy supply of the cell, but their derivatives also play some role in signal transfer between cells [82,83]. Collectively, these results suggest that patients with autoimmune diseases have abnormal lipid metabolism, and the function and status of macrophages in those patients should garner much attention as one of the major contributors to lipid metabolism. Furthermore, short-chain fatty acids (SCFAs) may potently act as a novel anti-inflammatory treatment for systemic inflammation [84].

### 4.4. Changes in Macrophage Mitochondria in the Injured Kidney of Autoimmune Disease

Mitochondria, known as the “powerhouse” of the cell, are energy-producing structures and the main sites of aerobic respiration in cells. Therefore, the quality of mitochondria is essential for cellular homeostasis and the maintenance of kidney function [85]. Many factors that influence the function of mitochondria will impact cellular homeostasis and the maintenance of kidney function. Dysfunctional mitochondria bring about kidney fibrosis by increasing macrophage oxidative stress [86]. Bhargava et al., indicated that macrophage function is controlled by mitophagy via the PINK1/MFN2/Parkin-mediated pathway and plays a protective role against kidney fibrosis. The dysregulation of either Pink1 or Prkn motivates renal extracellular matrix formation and profibrotic/M2 macrophage frequency [87]. As the primary organelle of cell metabolism, it is particularly necessary to attach importance to the structure and function of mitochondria in macrophages in autoimmune diseases. The study of mitochondria in macrophages may provide a new perspective for the treatment of kidney damage caused by autoimmune diseases by targeting macrophages.

### 4.5. Other Factors Contributing to Altered Macrophage Metabolism in the Injured Kidney of Autoimmune Disease

The binding of IgG antibodies to self-antigens leads to organ damage, which is the direct cause of kidney damage in systemic lupus erythematosus (SLE) and other autoimmune diseases. In recent research, interferon (IFN) regulatory factors 5 (IRF5) gene polymorphisms have been found to play an important role in human serum autoimmune disorders and may be associated with the development of typical clinical autoimmune diseases, which is largely dependent on the presence of anti-autoantibodies [88,89]. Typically, IRF5 as a member of the IRFs family is recognized as playing pivotal roles in the regulation of many facts of innate immune responses, which could enhance the response to environmental infectious factors [90]. In addition, the immune complex (IC) can increase inflammatory reactions the through IRF5/NF-κB pathway. Furthermore, IRF5 exhibits roles in the regulation of proinflammatory cytokines for the development of TH1 and TH17 responses, which also stimulate immune responses [91]. With the accumulation of IC, tissue-resident leukocytes such as macrophages show proinflammatory effects activated by antigen–antibody complexes through Fcγ receptors (FcγRs) ligating and other-inflammatory story factors. Further research found that glycolytic genes in macrophages were upregulated in response to IgG IC stimulation [92]. This metabolic reprogramming of macrophages directly induces the release of several proinflammatory mediators and cytokines in lupus nephritis and exacerbates the progression of kidney damage.

## 5. Macrophages in Autoimmune Disease Kidney Damage and Repair

Patients with autoimmune diseases have a chaotic immune system, and macrophages are the first line of defense in immunity. On the one hand, innate immune cells, including macrophages, are already well trained for certain stimuli to respond more strongly and activate the immune system to keep our body safe. On the other hand, the immune tolerance characteristics of macrophages help maintain immune balance [93]. In autoimmune diseases, macrophages, in the same way as trucks that have lost their brakes, continuously respond to proinflammatory factors in the microenvironment and amplify these signals. These signals can be devastating to the kidneys. Some of the chemokines such as C-C Motif Chemokine Receptor 2 (CCR2) and CCR5 could induce glomerular macrophage infiltration and contribute to the development of glomerular endocapillary hypercellularity in lupus nephritis [94]. In diabetic nephropathy (DN), the activation of M1/M2 through the Trem1 pathway is positively correlated with the progression of DN [95]. Even exosomes from high glucose-treated macrophages could activate glomerular mesangial cells via the TGF-β1/Smad3 pathway and cause tissue damage [96]. Here we summarize the role of macrophages in renal damage caused by different autoimmune diseases, paying more attention to the potential therapeutic role of macrophage metabolism intervention.

### 5.1. Lupus Nephritis and Macrophages

Systemic lupus erythematosus (SLE) is an autoimmune-mediated diffuse connective tissue disease characterized by immune inflammation and is a serious and potentially fatal autoimmune disease known as “immortal cancer” [97]. Kidney biopsies showed that almost all patients with SLE had different degrees of renal pathological changes. Between 2009 and 2016, macrophage activation syndrome (MAS) was observed in 9.62% of Moroccan adult patients with SLE in a retrospectively analyzed study from the Department of Internal Medicine [98].

The response of macrophages to inflammation is based on the different signals they receive, and the functions of macrophages are orchestrated by the synergistic effect of multiple stimuli. Autoimmune diseases, especially SLE, are caused by a dysregulated immune system that will produce large amounts of autoantibodies [99]. Research based on SLE patients demonstrated that microparticle-immune complexes (MP-IC) from patients with SLE favored the polarization of monocyte-derived macrophages (MDM) into a proinflammatory profile that promotes IFN-γ^+^CD4^+^, TNF-α^+^CD4^+^, and IFN-γ^+^CD8^+^ T-cell increase, and additionally induces B-cell activation and survival. These results showed how macrophages play an amplifier role in SLE [100]. At the same time, the impaired function of Treg cells, which act as brakes, reduces the regulation of the immune system, thus promoting the progression of the disease [101,102]. Moreover, Fc is an integral part of naturally occurring antibodies. Correspondingly, Fc is recognized by the Fc receptor. Abundant Fcγ receptors (FcγRs) expressed on the surface of macrophages can respond to local immune changes and promote the production of many cytokines, such as IL-6, TNFα, and IL-1β, as well as inflammatory mediators. Additionally, the AhR activation worsened the disease severity in the deficiency of Fcγ receptor IIb (FcγRIIb), which is the only inhibitory FcγR in the FcγR family [103]. A recent report related to IC stimulation suggested that macrophages upregulate glycolytic genes and undergo a switch to aerobic glycolysis [104]. By using single-cell RNA sequencing (scRNAseq), researchers found that fatty acid metabolism genes were enriched in no diseased MRL/MpJ kidney monocytes and macrophages; however, glycolysis and OXPHOS genes were increased in MRL-lpr mice, which is the M1 metabolism feature. More specifically, two inflammatory responses associated with the extracellular acidification rate (ECAR) and oxygen consumption rate (OCR) were changed in macrophage metabolism. Moreover, another group found that IFN-γ, as a product of inflammatory response, also acts on the regulation of macrophages. Under normal physiological conditions, there is a negative feedback mechanism for proinflammatory IFN-γ activation in macrophages, and macrophage-associated matrix metalloproteinase 12 (MMP12) can efficiently remove the IFN-γ receptor-binding site by truncating the C-terminal of IFN-γ. Therefore, proinflammatory macrophage activation is reduced. However, compared to treated patients with healthy individuals, the MMP12 levels were lower and IFN-γ were higher. These studies are in line with previous studies. In the publication, the researcher used two types of SLE mouse models, MRL/MpJTnfrsf6lpr and NXBW/F1. After first being immunized against a small immunogenic hapten, the mouse was treated with folate-linked fluorescein. Once macrophages are recognized by anti-fluorescein antibodies, they are the casing for macrophage removal [105]. Macrophage clearance extended the lifespan of both models. Together, these results show that macrophages are potential therapeutic targets in lupus nephritis.

GW2580, which is a kinase inhibitor monospecific for the colony-stimulating factor-1 (CSF-1), is used to clear macrophages. Their data showed that the preventive administration of GW2580 in nephrotoxic serum (NTS) ameliorates renal function and inhibits histopathological kidney damage, suggesting that the deletion of macrophages is beneficial for nephritis improvement [106]. Many researchers have found that macrophages may be regulated by metabolites that alter the inflammatory state, highlighting that we can mediate the role of macrophages in autoimmune diseases by regulating metabolism-related substances. Virgin olive oil (VOO) contains high levels of unsaturated fatty acids and has anti-inflammatory, antioxidant, and antiproliferative activities. VOO exerts immune regulatory activity both in human primary monocytes and monocyte-derived macrophages and in a pristane-systemic lupus erythematosus model, indicating that some unsaturated fatty acids will be helpful in the management of SLE [107]. Several metabolism-related drugs have potential effects in the treatment of SLE. The results showed that mycophenolate (MPA) and cyclosporin A (CsA) are two immunosuppressants currently used to treat autoimmune diseases that could regulate lipid metabolism and cholesterol influx, especially in different subtypes of macrophages [108].

Collectively, metabolic regulation of macrophages in lupus erythematosus patients can alleviate disease progression and provide some protection against kidney damage [109,110]. This study provides a new direction for the treatment of LN.

### 5.2. Diabetic Nephropathy and Macrophages

Type 1 diabetes, also known as insulin-dependent diabetes, is a metabolic disorder syndrome [111]. There are no studies confirming the definitive cause of type 1 diabetes. Researchers have found that type 1 diabetes is an autoimmune disease. A variety of autoimmune antibodies such as glutamic acid decarboxylase antibodies (GAD antibodies), and islet cell antibodies (ICA antibodies) can be found in the blood of patients with type 1 diabetes. The possible influence of environmental factors such as bacterial and viral infections could also be linked to the development of type 1 diabetes because the GAD molecule has a similar amino acid sequence to proteins from sources such as cytomegalovirus, coxsackie virus, and adenovirus, etc.; pancreatic β-cells may be misidentified and killed by the immune system when infected [112]. Toll-like receptors (TLRs) play an important role in innate immunity. They act as the eyes of innate immunity, monitoring and recognizing various disease-associated molecular patterns (PAMP), and are the body’s first barrier against infectious diseases. However, lymphocytes cross-react due to the homology of viruses and endogenous antigens, as well as genetic predisposition to make the target organs vulnerable to attack by autoimmune cells. The immune balance is disrupted accompanied by secreting autoantibodies, which eventually leads to diseases [113]. These abnormal autoantibodies can damage pancreatic islet B cells leading to insulin secretion. Diabetes nephropathy is one of the most common complications in diabetic patients. Similar to type 1 diabetes, the etiology and pathogenesis of diabetic nephropathy are not believed to be caused by a single factor; rather, they are caused by the joint action of certain genetic backgrounds and some risk factors [114,115]. In the diabetic state, glucose metabolism disorders occur in all organs of the body, including the kidney, nerve, eye, and other tissues/organs. At this time, approximately 50% of glucose is metabolized in the kidney, which reduces the risk of ketoacidosis and hypertonic coma. However, a high metabolic level will aggravate the glucose metabolism in the kidney and change the metabolism of macrophages [116]. There is a complex interaction network between the innate immunity complement system and pattern recognition receptors, which play an important role in the pathogenesis of diabetic nephropathy [117]. In addition, macrophages and mast cells, various transcription factors, chemokines, adhesion molecules, inflammatory factors, and glycosylated metabolic final products may participate in pathogenic mechanisms [118]. Among them, macrophages may become an important intervention target in diabetes nephropathy processes [119,120,121].

Macrophage polarization is strongly linked to glomerular destruction and fibrosis in type 1 diabetes. Fasudil, as a Rho-associated coiled-coil containing protein kinase (ROCK) inhibitor, could retard renal interstitial fibrosis by regulating macrophage polarization [122]. The sodium-glucose cotransporter 2 (SGLT2) inhibitor dapagliflozin suppressed inflammation, and oxidative stress, and reduced macrophage infiltration into the glomeruli and interstitium [123]. These results suggest that macrophage infiltration may be reduced by alleviating kidney inflammation, thereby attenuating the progression of diabetic nephropathy. Studies have examined whether type 1 diabetes and diabetic nephropathy can be alleviated if macrophages are used as target cells. Cyclooxygenase 2 (Cox-2) is an induced enzyme, and the expression of COX-2 in normal tissue cells is very low. When Cox-2 is stimulated by inflammation, the expression of COX-2 in inflammatory cells increases 10–80 times. COX-2 is also expressed in renal immune cells, especially renal monocytes/macrophages. However, activated COX-2 induces macrophage polarization to the M2 phenotype through the PGE2-EP4 axis and protects against the development of diabetic nephropathy. Although these results go against common sense, they also illustrate the complex mechanisms of kidney damage in autoimmune diseases [124,125]. Kidney injury is influenced by the mechanisms of renal injury, the expression and subtype of receptors, and the timing of treatment. However, the role of macrophages in different phenotypes is relatively constant; M1 promotes damage, while M2 plays a protective role.

Several therapeutic strategies have been proposed for macrophages to treat different diseases. The administration of mesenchymal stem cells (MSCs) from human umbilical cord blood could promote M2 polarization by upregulating arginase-1 (Arg1) to improve mitochondrial biogenesis and function in tubular epithelial cells (TECs) [126,127]. These effects may halt further inflammation and decline in kidney function. Amazingly, damaged macrophage mitochondria are replaced with MSCs and exogenous healthy mitochondria, which contribute to dampening inflammation and kidney injury in DN mice. In vitro, MSCs-Mito was found to change energy metabolism, and inhibit the inflammatory response [128]. When MSCs-Mito was transferred into RAW264.7 cells, the results suggested that regulating the metabolic status of macrophages has a certain therapeutic role in autoimmune diseases. Transforming growth factor β-activated kinase 1-binding protein 1 (TAB1) can specifically combine with transforming growth factor β-activated kinase 1 (TAK1), which conduces macrophage activation related to aggravated DN [129]. Its complex interacts with HIF-1α through nuclear factor κB (NF-κB). The expression of IL-1β and MCP-1 secreted from bone marrow-derived macrophages (BMMs) decreased after interference with TAB1. Confocal laser scanning showed that the glycolytic enzymes hexokinase-1 (HK1), 6-phosphofructo-2-kinase/fructose-2 (PFKFB), 6-biphosphatase 3 (PFKFB3), and lactate dehydrogenase A (LDHA) were reduced when TAB1 expression was silenced [130]. This study proposed that DN inflammation can be improved by mediating glycolysis and the polarization of macrophages. Moreover, some studies about the pathways of amino acid metabolism and lipid metabolism have also shown some surprising results, suggesting that the effects on macrophage metabolism may have unexpected effects in the treatment of autoimmune diseases [131,132,133,134,135]. All these new findings offer hope for diabetics to fight the disease.

### 5.3. Systemic Sclerosis and Macrophages

Systemic sclerosis (SSc) is an autoimmune disease characterized by immune dysfunction, fibrosis, and vasculopathy [136]. There is evidence that macrophages are involved in scleroderma fibrosis and the release of inflammatory factors. Renal fibrosis due to scleroderma is insidious and extensive in contrast to the obvious skin lesions. Occult renal pathology occurs in 60–80% of SSc patients [137]. For instance, M2 macrophages play a role in promoting fiber development during scleroderma through derived growth factors and have effects on monocyte chemotaxis and collagen synthesis [138]. Some studies have shown that the progression of lung injury and fibrosis is alleviated by inhibiting the polarization of M2 macrophages. However, M1 can induce the release of inflammatory factors, which is not conducive to the development of the disease [139]. Recently, the concept of “trained immunity” has emerged as a new dawn to change the function of macrophages. It is generally believed that some immune cells differentiate into memory cells after receiving antigen stimulation, then produce a rapid and efficient response to the re-accepted antigen, to achieve the effect of the rapid elimination of pathogens [140,141]. In contrast, a small amount of stimulation may blunt the macrophage response, which means that we may greatly reduce the inflammatory state by educating macrophages in autoimmune patients to be insensitive to the microenvironment in their body, allowing macrophages to remain normal so that there is no excessive repair or development of fibrosis [142,143,144,145]. Research has shown dampening of the fibrotic phenotype in the skin and visceral organs cocultured with PCG vaccine and low-dose LPS-trained human macrophages, suggesting that the macrophages in the patient can have an increased threshold for stimulation in the microenvironment without losing their normal functions [146,147,148]. This finding indicates a new potential treatment for autoimmune diseases that do not cause hypercorrection.

### 5.4. Renal Injury Induced by Other Autoimmune Diseases and Macrophages

Rheumatoid arthritis (RA) is also a clinically common chronic autoimmune disease, dominated by inflammatory synovitis [149,150]. Although the specific mechanism is still controversial, it is undeniably an autoimmune disease, RA is also the result of the joint action of environmental pathogenic factors and self-susceptibility genes. The patient’s immune system is dysfunctional based on a number of different genetic factors, combined with environmental factors such as HERV-W/K, Mycobacteria, and EBV infection [151]. Although RA is characterized by chronic erosive polyarthritis, it can induce kidney damage; some kidney failures are caused by anti-inflammatory medications in the treatment, and others are induced by RA itself. The angiotensin II/AT1 pathway may be related to renal injury induced by rheumatoid arthritis. The number of macrophages around renal tubules increases in parallel with angiotensin receptor subtype 1 (AT1) expression, and the synergistic effect can be impacted by the intervention of losartan, which is an angiotensin-converting enzyme (ACE) or AT1 inhibitor [152,153]. R.L. Papke demonstrated that the cholinergic anti-inflammatory pathway (CAP) plays a critical role in the pathogenesis of RA. α7 nicotinic acetylcholine receptors (α7nAChRs) as a part of the CAP pathway are expressed at varying levels in all types of immune cells, including macrophages, and have shown potential anti-inflammatory effects [154]. These phenomena suggest that during the treatment of rheumatoid arthritis, it is necessary to evaluate the inflammatory state of patients macroscopically and comprehensively to consider the benefits of treatment. The patient’s renal function must be paid attention to over time, with intervention when necessary, or renal protection drugs given while treating rheumatoid arthritis. In addition, targeted delivery of anti-inflammatory medications that encapsulate the drug into a high-molecular polymer such as PLGA-PEG NPs appears to be a promising strategy to reduce finite side effects and improve the drug utilization rate [155]. However, this method remains controversial because it only focuses on a single factor and cannot completely avoid damage to other tissues. It should be noted that in recent years, studies have found that cell function can also be regulated by adding some additional cellular metabolites.

## 6. Conclusions

As part of the immune “junction center” and “immunological surveillance sentry”, the plasticity and heterogeneity of macrophages determine their roles in the inflammatory response. They play diverse roles in different diseases and different courses of the same disease. Especially in autoimmune diseases, under the influence of the complex immune microenvironment, macrophages may even affect the progression of the disease from two opposite directions. At different pathological stages of renal fibrosis, macrophages can exhibit dual proinflammatory and anti-inflammatory effects. Therefore, with the help of advanced technologies, such as single-cell sequencing, a more comprehensive analysis of macrophages can be carried out to guide the establishment of a clinical treatment scheme by analyzing the characteristics and functions of each cell type. It is worth noting that macrophages are not only complex immune cells; as a part of the immune regulatory network, the simple pursuit of increasing or decreasing macrophages is not necessarily a good solution. Combined with recent research on metabolism, we believe that pursuing a relative balance and stability is a new direction for the treatment of autoimmune diseases in the future. Macrophages play a decisive role in this process.
